# Facial emotion recognition in adopted children

**DOI:** 10.1007/s00787-021-01829-z

**Published:** 2021-07-06

**Authors:** Amy L. Paine, Stephanie H. M. van Goozen, Daniel T. Burley, Rebecca Anthony, Katherine H. Shelton

**Affiliations:** 1 School of Psychology, Cardiff University, Tower Building, 70 Park Place, Cardiff, CF10 3AT UK; 2 Wolfson Centre for Young People’s Mental Health, Cardiff University; Centre for the Development and Evaluation of Complex Interventions for Public Health Improvement (DECIPHer), School of Social Sciences , Cardiff University, 1-3 Museum Place, Cardiff, CF10 3BD UK

**Keywords:** Adoption, Emotion recognition, Emotional problems, Behavioural problems

## Abstract

Children adopted from public care are more likely to experience emotional and behavioural problems. We investigated two aspects of emotion recognition that may be associated with these outcomes, including discrimination accuracy of emotions and response bias, in a mixed-method, multi-informant study of 4-to-8-year old children adopted from local authority care in the UK (*N* = 42). We compared adopted children’s emotion recognition performance to that of a comparison group of children living with their birth families, who were matched by age, sex, and teacher-rated total difficulties on the Strengths and Difficulties Questionnaire (SDQ, *N* = 42). We also examined relationships between adopted children’s emotion recognition skills and their pre-adoptive histories of early adversity (indexed by cumulative adverse childhood experiences), their parent- and teacher-rated emotional and behavioural problems, and their parents’ coded warmth during a Five Minute Speech Sample. Adopted children showed significantly worse facial emotion discrimination accuracy of sad and angry faces than non-adopted children. Adopted children’s discrimination accuracy of scared and neutral faces was negatively associated with parent-reported behavioural problems, and discrimination accuracy of angry and scared faces was associated with parent- and teacher-reported emotional problems. Contrary to expectations, children who experienced more recorded pre-adoptive early adversity were more accurate in identifying negative emotions. Warm adoptive parenting was associated with fewer behavioural problems, and a lower tendency for children to incorrectly identify faces as angry. Study limitations and implications for intervention strategies to support adopted children’s emotion recognition and psychological adjustment are discussed.

## Introduction

Every child adopted from state care has a complex and individual history of early adversity [1, 2] that may include early experiences of maltreatment, neglect, or household dysfunction. Some children may have been exposed to drugs or alcohol *in ute*ro, or may have a higher genetic risk for mental health problems [3, 4]. All children placed for adoption experience separation from their birth parents and some degree of instability in their living circumstances during their time in care [5]. Although the context of the adoptive family aims to provide children with stability, protection, and a loving and stimulating environment, adverse early experiences can have profound consequences for development [6, 7]. Although evidence suggests that many adopted children fare well, their experiences of early adversity places them at greater risk for developing emotional and behavioural problems that may endure in the years after they come to live with their adoptive family [8–12]. Developing knowledge of adoptees’ cognitive and affective development has the potential to provide targets for effective intervention that support the adjustment of adopted children and their families [1, 2]; however, studies that investigate specific domains of development of children adopted from public care are scarce (for exceptions see [13, 14]).

## Emotion recognition

Understanding emotions is fundamental to effective social relationships and psychological adjustment [15]. Visual recognition of emotions from facial cues is a vital component of children’s ability to effectively navigate social interactions [16], and includes two distinct processes: discrimination accuracy and response bias [17]. Discrimination accuracy refers to the ability to correctly identify emotions, whereas response bias indicates the tendency to attribute emotions in the absence of concordant cues (i.e., mistakenly identifying non-hostile, ambiguous or neutral expressions as angry), which can potentially reveal patterns of errors in children’s attributions of emotions [18]. Good recognition of emotional expressions from facial cues is associated with positive social relationships, social competence, and peer acceptance [19]. Conversely, impairments in emotion recognition are associated with social rejection, victimisation, and adjustment problems and clinical symptoms in childhood [20–22]. For example, poor recognition of emotions is associated with emotional problems [23] and deficits in recognizing specific negative emotions, such as sadness, fear, and anger, are evident in children with behavioural problems [24]. Studies demonstrate that children with emotional and behavioural problems may be hypersensitive to anger [22, 25, 26], and impaired recognition of fearful expressions is implicated in children with behaviour problems [16, 27]. As such, children with emotional and behavioural problems may struggle with everyday social insight that guides behaviour in emotionally-charged situations [28, 29].

## Early experience

Early experience plays an important role in the development of how children perceive emotion [29]. Risk factors in the prenatal period (e.g., exposure to mothers’ substance use in utero) and experience of adversity in early life, such as maltreatment, neglect, and household dysfunction are known to affect neuropsychological systems associated with children’s emotional perception [24]. Early socialisation also has long-term implications for children’s recognition of emotion [31, 32] and children who experience adverse rearing conditions may have different profiles of emotion recognition accuracy and bias [33]. For example, children who experience maltreatment display superior recognition of anger and a hostile bias compared to non-maltreated children [34, 35], possibly because they are more likely to experience negative and hostile outbursts from their caregiver, and interactions that are frightening and inconsistent [36]. In contrast, children who experience few or impoverished opportunities for emotional learning in neglectful rearing conditions show greater difficulty discriminating emotional facial expressions [35, 37]. In extreme cases of privation, children raised in institutional caregiving facilities (i.e., with a lack of social stimulation, toys, and individualised care) show difficulty identifying facial expressions of distinct emotions [38, 39].

Although previous research suggests that an adverse early social environment has a lasting impact on the development of emotion recognition, studies of non-adopted and post-institutionalised children cannot be used to make inferences about the development of children adopted from state care [40]. Children who are taken into local authority care (looked after; fostered) in the UK are removed from their birth family when their parent(s) are unable to care for them; this is most often in cases of abuse or neglect [41]. Following their removal, most children spend some time in temporary kinship care or with foster carers prior to their adoption [42]. Although such intervention by the state removes the immediate risk of harm, some children experience uncertainty and instability in their living arrangements until they come to live with their adoptive family [5]. Evidence from US and UK samples indicate that care-experienced and children adopted from foster care have higher rates of mental health problems than non-adopted children [43–46] and children adopted via other pathways (e.g. private adoptions, [47]). Children adopted over the age of 4 show the most problems [46], quite possibly because they are more likely to have experienced pre-placement adversity [48] and multiple placements—and therefore greater instability—while they were a ward of the state [49]. Although carers have reported emotional processing and a hostile bias as relevant explanations for looked after children’s adjustment problems [50], little is known about emotion recognition in children adopted from state care, and associations with pre-adoptive early experiences and post-adoptive psychological adjustment.

## Adoptive parenting

The family environment offered by adoptive parents is an influential factor in a child’s psychological adjustment [51]. Children of adoptive parents who exhibit warm, sensitive, and responsive parenting demonstrate a marked reduction in emotional and behavioural problems [9, 52], quite possibly because warm parenting fosters emotional competence and models positive social interactions that shape a child’s behaviour in other relationships [53]. The emotional tone of the family environment is also well known for its role in children’s development of emotional understanding [23, 54–56] and parents’ emotion-related beliefs, behaviours, skills, supportive parenting are predictive of emotion recognition in childhood [47, 58]. Adoptive family life that is characterised by warmth may provide children with opportunities to make sense of the social world and explore positive and negative emotions in a safe context. However, relatively few studies have examined the role of warm parenting on children’s recognition of emotion (for an exception, see [32]), and to our knowledge, no studies to date have examined emotion recognition in children adopted from local authority care, and associations with their emotional and behavioural problems and the emotional environment offered by adoptive parents.

## The present study

Although a child’s circumstances are dramatically altered through adoption, children adopted from state care in the UK are likely to have experienced a cluster of pre-adoptive adverse experiences that place them at higher risk for emotional and behavioural problems [9, 11]. Understanding of specific domains of development has the potential to inform targets for intervention [13, 14], and these targeted interventions may be enhanced by knowledge of the emotion recognition abilities of children adopted from local authority care. We addressed this problem by investigating facial emotion recognition in a mixed-method, multi-informant study of 4-to-8-year-old children adopted from local authority care in the UK.

The current study had four aims, to: 1) investigate adopted children’s emotion recognition as it compares to children living with their birth families. We compared adopted children’s emotion recognition to non-adopted children at similar risk for future psychopathology to elucidate the impact of early adversity and adoptive experience on adopted children’s emotion recognition skills. We also examined associations between adopted children’s emotion recognition and 2) parent and teacher ratings of their emotional and behavioural problems; 3) their history of early adversity, indexed by a number of cumulative adverse childhood experiences [6, 43]; and 4) adoptive parents’ warm parenting. By collecting and integrating information regarding children’s emotional and behavioural problems from multiple informants, we were able to investigate associations between children’s emotion recognition and their behaviour in different contexts, and capture whether associations were consistent across informants.

We hypothesised that compared to non-adopted children, adopted children would exhibit a hostile bias (i.e., have a greater likelihood to mistakenly label non-angry faces as angry). Given that accuracy of discriminating distinct emotions differs according to the history of adverse rearing conditions (maltreatment or neglect; [34]), we made no predictions regarding adopted discrimination accuracy compared to non-adopted children.

We hypothesised that children who experienced more pre-placement adverse experiences would exhibit a greater bias towards negative emotions. We also hypothesised that low discrimination accuracy of negative emotions (sadness, fear, and anger) and a hostile bias would be positively associated with emotional and behavioural problems. Finally, we hypothesised that warmth exhibited by adoptive parents would be associated with better emotion recognition in their children. In our analyses, we investigated covariates of emotion recognition, given that emotion recognition is known to vary as a function of age, sex, and language ability in childhood [20, 23, 59].

## Methods

### Design

The study included 84 children aged 4 to 8 years who were assessed at the Neurodevelopment Assessment Unit (NDAU) (https://www.cardiff.ac.uk/neurodevelopment-assessment-unit). The NDAU provides the setting for a feasibility study of an innovative and rigorous approach to the assessment and characterisation of neurodevelopmental problems in children. Children and a parent are invited to the NDAU following a referral from their school teacher where challenges are identified. Ethical permission was granted by Research Ethics Committees at Cardiff University (School of Psychology) Research Ethics Committee and the study was performed in accordance with the ethical standards laid down in the 1964 Declaration of Helsinki and its later amendments. Written informed consent was obtained from a parent or caregiver for each child who participated in the assessment.

### Participants

The present study focused on a sample of adopted children who took part in the facial emotion recognition task during their assessment visit at the NDAU (*N* = 42, 23 female, *M* age = 78.12 months, *SD* = 11.87). Eleven adopted children (26.2%) were referred to the NDAU by their school teacher for a range of socioemotional, behavioural, and cognitive difficulties. Thirty-one (73.8%) who were eligible for referral (aged between 4 and 7 at the time of recruitment) were invited to attend an assessment via their own or their sibling’s participation in the Wales Adoption Cohort Study; a prospective longitudinal study of 96 children placed for adoption from care in Wales between 1st July 2014 and 31st July 2015 (see [9] for more details of the study [4, 9] for background of adoption in the UK). School teachers provided equivalent referral documents for each child.

The 42 adopted children in the present study were placed for adoption at a median age of 2 years (*IQR* = 1 to 3), having spent a mean of 277.26 (*SD* = 369.27) days with their birth parents. Children spent *M* = 424.95 (*SD* = 172.46) days in local authority care, and experienced a median of 2 moves (*IQR* = 1 to 4) between homes whilst in care. Most adoptive parents were white British (*N* = 40, 95.2%), in full- or part-time work (*N* = 33, 80.5%), *N* = 30 (71.4%) had a university degree or higher, and *N* = 11 (26.2%) earned more than £60,000 a year which was higher than the UK average according to Office for National Statistics data [60]. In terms of adopted children’s histories of early adversity, for the 31 of 42 children who participated in the neurodevelopmental assessment as part of their participation in the Wales Adoption Cohort Study, pre-adoptive adverse experiences were retrieved from social worker records. Adoptive parents also reported on their child’s experiences during the interview at the NDAU. We used information from pre-placement social work records and evidence from the parent interviews to ascertain the number of adverse childhood experiences the children experienced prior to their adoptive placement. These experiences included any evidence of the presence of 10 categories of ACEs (see [6, 43]), including abuse (emotional, physical, or sexual), neglect, and household dysfunction (domestic violence, parental separation, substance abuse, alcohol abuse, mental illness, or incarceration); the adopted children experienced a median of 1 (*IQR* = 0 to 3) adverse childhood experiences prior to their adoptive placement.

To investigate the generalisability of the adopted sample to their population, we compared the child and parent characteristics of the children who were assessed in NDAU with children taking part in the Wales Adoption Cohort Study who were eligible for inclusion and with the population of interest (all children adopted between 1st July 2014 and 31st July 2015). Except for child age (where children in the present study were younger than the population of interest at the time of adoption) the adopted children and their families were representative of the population from which they were drawn (see [13]).

We also used data from a comparison sample of non-adopted children living with their birth parents (*N* = 42, 23 female, *M* age = 78.14 months, *SD* = 11.92). The non-adopted comparison sample were matched to the adoption sample by age (within 6 months), sex, and within the same band according to the 4-band categorisation of teacher-rated total difficulties according to the Strengths and Difficulties Questionnaire (SDQ; close to average, slightly raised, high, or very high; [61]). Table 1 describes the sociodemographic and child characteristics of the adoption and non-adoption samples. The 42 matched non-adopted children were comparable to the adoption sample in terms of ethnicity, parent education, and employment status (all *p*s > 0.10). There was some imbalance detected between groups in terms of household income, with the adoption sample containing higher earners than the comparison sample, χ(3) = 9.12, *p* = 0.03. No significant differences were detected for child characteristics following matching (age, sex, SDQ total difficulties, and receptive vocabulary, all *p*s > 0.05).

**Table 1:** Sample characteristics

	Adoption sample (*N* = 42)	Matched non-adopted comparison sample (*N* = 42)
Family characteristics		
Household income^a^ (*N*, %)		
Up to £19,999	1 (2.4)	10 (24.4)
£20,000 to £39,999	12 (29.3)	12 (29.3)
£40,000 to £59,999	17 (41.5)	11 (26.8)
£60,000 +	11 (26.8)	8 (19.5)
Primary caregiver ethnicity (*N*, %)		
White British	40 (95.2)	38 (90.5)
Primary caregiver education (*N*, %)		
No formal educational qualification	0 (0.0)	2 (4.8)
O-Levels or GCSEs	7 (16.7)	8 (19.0)
A Levels/Higher	5 (11.9)	10 (23.8)
University degree	11 (26.2)	11 (26.2)
Higher or postgraduate degree	19 (45.2)	11 (26.2)
Primary caregiver employment^a^ (*N*, %)		
Full time paid employment	14 (34.1)	12 (28.6)
Part time paid employment	19 (46.3)	17 (40.5)
Not in paid employment	8 (19.5)	13 (31.0)
Parental warmth (*M, SD*)	3.45 (0.90)	3.38 (1.01)
Child Characteristics		
Sex (*N*, % female)	23 (54.8)	23 (54.8)
Age at assessment (*M, SD*)	78.12 (11.87)	78.14 (11.92)
Total difficulties^b^ (*M, SD*)	12.17 (6.68)	13.17 (6.47)
Verbal ability^c^ (*M, SD*)	91.30 (12.09)	96.14 (12.40)

^a^Some missing data as questionnaire partially completed

^b^Teacher ratings on the Strengths and Difficulties Questionnaire (SDQ); teacher ratings of total problems that range between 12 and 15 indicate slightly raised total difficulties [61]

^c^Age normed standardised scores on the British Picture Vocabulary Scale (BPVS; [67])

### Procedure

The children and a parent were invited to the NDAU for two assessment sessions; the first for 3 h, the second for 2 h. Following a short introduction together, the child completed assessments in the testing room with a trained developmental assessor. Assessments included a battery of well-established tasks used internationally in research and clinical practice targeting underlying dimensions of functioning, based on the Research Domain Criteria (RDoC) approach; a research framework to investigate mental disorders by measuring domains of functioning (emotion, cognition, motivation, and social behaviour) (https://www.nimh.nih.gov/research/research-funded-by-nimh/rdoc/index.shtml). The child’s parent completed an interview and questionnaires in a separate interviewing room.

### Measures

#### Facial emotion recognition

Children’s emotion recognition was assessed using the Facial Emotion Recognition task (FER; [62, 63]). Children were presented with 40 faces depicting happy, sad, fearful, angry, and neutral expressions (Radboud Faces Database; [64]). Four low, and four high-intensity versions of the affective expressions were presented; intensity versions were created by merging a 100% intensity target expression with a neutral expression, which was validated by independent raters [62]. Each facial expression was presented on a laptop for 3 s, before the emotion category labels appeared in the text on the screen, and the child was asked to identify the facial expression verbally with no time limit applied for their response. The presentation order of the facial expressions was pseudo-randomized across two task versions.

We used the two-high threshold (2HT) approach [65] to transform the raw data from the facial emotion recognition task to produce discrimination accuracy (Pr) and response bias (Br) variables for each emotion and neutral faces. This approach has been previously used to examine emotion recognition accuracy in children [20, 66]. Discrimination accuracy represents children’s ability to discriminate amongst target emotional and neutral faces, and response bias represents children’s tendency to incorrectly categorise emotional and neutral faces as a certain emotional expression or as neutral. This approach is based on the computation of hit rates (HR), using hits (H) and targets (T), and false alarm rates (FAR), using false alarms (F) and distractors (D), with simple transformations to produce Pr and Br variables using the following formulas:

$$HR=\frac{H+0.5}{T+1}$$

$$FAR=\frac{F+0.5}{D+1}$$

$$Pr=HR-FAR=\frac{H+0.5}{T+1}-\frac{F+0.5}{D+1}$$

$$Br=\frac{FAR}{1-Pr}$$

#### Emotional and behavioural problems

Adoptive parents and teachers completed the Strengths and Difficulties Questionnaire (SDQ; [61]). We used internalising (sum of emotional and peer problem scales) and externalising problem (sum of conduct and hyperactivity scales) total scores. A higher score is indicative of more problems for both subscales (where children could score a maximum of 20 for each scale). Internalising and externalising subscales had acceptable to good levels of internal consistency for parent (αs = 0.83 externalising and 0.78 internalising) and teacher reports (αs = 0.83 externalising and 0.74 internalising).

#### Receptive vocabulary

Children’s receptive vocabulary was assessed using the British Picture Vocabulary Scale (BPVS; [67]). In each trial of the test, children were presented with four pictures. The experimenter said one word aloud, and child was asked to select the picture that matched with the meaning of the word. Children received two practice trials where feedback was given if incorrect. The task was terminated after children exceeded a predefined threshold of errors. Age-normed standardised scores were calculated.

### Parental warmth

Parental warmth was assessed using a Five-Minute Speech Sample (FMSS; [68]). During this task, parents (97.6% mothers) were asked to describe their child’s personality and their relationship for five minutes. Parents were not prompted unless they were struggling to continue talking, in which instance they were given set probes by the researcher such as, “How would you describe [child’s] personality?” Each parent speech sample was recorded and transcribed. Independent coders, who were blind to the aims of the study, rated the content of the sample and tone of voice when describing their child for warmth, using a rating of 0 (no warmth) to 5 (high warmth) using guidelines described in [69]. A subsample of 15/84 (17.9%) videos were coded by three additional independent raters, *ICC* = 0.95. Independent samples *t-*tests indicated no differences between the adopted and non-adopted groups in parental warmth (*p* = 0.75).

### Data analysis

Our first aim was to compare adopted children’s discrimination accuracy and response bias in the emotion recognition task with non-adopted matched children. Comparisons were investigated using ANOVA-based methods. Kolmogorov–Smirnov tests of normality indicated standardised residuals for happy and angry discrimination accuracy and response bias scores were not normally distributed (all *p*s < 0.05). However, examination of Q-Q plots and histograms suggested distribution was acceptable and we proceeded with parametric tests given that ANOVA-based tests are robust to violations of normality. Partial eta-squares estimated the effect sizes for the ANOVA-based tests, where values of 0.02, 0.13, and 0.26 are indicative of small, medium, and large effect sizes, respectively [70]. Bonferroni correction was applied when assessing pairwise comparisons.

Aims 2 to 4 were to investigate associations between adopted children’s emotion recognition and their parent- and teacher-rated emotional and behavioural problems, their pre-adoptive history of early adversity, and their adoptive parents’ warmth during the 5-min speech sample. Associations were tested between variables of interest using Pearson or Spearman correlations according to the distribution of the data, partialling out identified covariates (i.e., sex and age at assessment). Analyses were considered statistically significant at *p* < 0.05.

## Results

### Emotion recognition in adopted and non-adopted children

Table 2 shows the means and standard deviations for discrimination accuracy and response bias between adopted children and non-adopted children in the comparison sample. MANOVA was used to examine differences in discrimination accuracy according to group (adopted vs. non-adopted comparison). There was a significant main effect of group on discrimination accuracy, Wilks’ *λ* = 0.87, *F*(5,78) = 2.41, *p* = 0.04, *η*_p_^2^ = 0.13. Pairwise comparisons showed that children in the adoption sample had lower discrimination accuracy for sad, *M* difference = −.08, 95% *CI*[− 0.18 to − 0.01], *p* = 0.03, and angry faces, *M* difference =  − 0.18, 95% *CI*[− 0.28 to − 0.07], *p* = 0.001. There was no effect of group on children’s response bias scores, *p* = 0.34.

**Table 2:** Mean (SD) discrimination accuracy and response bias for emotional and neutral faces for adopted and comparison samples

	Adoption sample (*N* = 42)	Matched non-adopted comparison sample (*N* = 42)
Discrimination accuracy (PR)		
Happy	0.80 (0.27)	0.83 (0.13)
Sad	**0.55 (0.20)**	**0.63 (0.16)**
Scared	0.50 (0.26)	0.60 (0.24)
Angry	**0.53 (0.26)**	**0.71 (0.21)**
Neutral	0.52 (0.22)	0.61 (0.23)
Response bias (BR)		
Happy	0.28 (0.17)	0.23 (0.16)
Sad	0.19 (0.17)	0.17 (0.19)
Scared	0.10 (0.10)	0.11 (0.11)
Angry	0.09 (0.11)	0.12 (0.11)
Neutral	0.53 (0.27)	0.59 (0.24)

Discrimination accuracy scores that tend toward -1 = worse than chance, 0 = chance, 1 = better than chance. Response bias scores that tend toward 0 = absence of bias, 1 = presence of bias. Significant differences between groups (where *p* < 0.05) are presented in bold

**Table 3:** Associations between adopted children’s emotion recognition scores and their parent- and teacher-rated emotional and behavioural problems and their history of pre-adoptive adversity

	1	2	3	4	5	6	7	8	9	10	11	12	13	14	15	16
1. PR happy	–															
2. PR sad	0.19	–														
3. PR scared	0.24	0.49**	–													
4. PR angry	0.36*	0.54**	0.59**	–												
5. PR neutral	0.64**	0.50**	0.62**	0.66**	–											
6. BR happy	0.02	0.13	− 0.02	0.13	− 0.14	–										
7. BR sad	0.03	0.20	− 0.13	-0.10	− 0.16	0.10	–									
8. BR scared	− 0.01	− 0.04	0.49	0.15	0.00	− 0.06	0.0	–								
9. BR angry	0.30^+^	0.17	0.30	0.58**	0.38*	0.07	0.23	0.23	–							
10. BR neutral	0.35*	0.03	0.10	0.08	0.48**	− 0.41**	− 0.58**	− 0.46**	− 0.26	–						
11. Parent emotional	0.26	− 0.26	− 0.21	− 0.35*	− 0.11	− 0.18	0.29^+^	− 0.07	0.06	− 0.10	–					
12. Parent behavioural	− 0.29^+^	− 0.18	− 0.45**	− 0.28^+^	− 0.41**	− 0.09	0.16	− 0.04	− 0.01	− 0.27^+^	0.46**	–				
13. Teacher emotional	0.06	− 0.20	− 0.38*	− 0.27^+^	− 0.17	0.03	0.15	− 0.22	0.00	0.01	0.59**	0.39*	–			
14. Teacher behavioural	− 0.17	− 0.08	− 0.24	0.03	− 0.16	− 0.06	− 0.22	− 0.06	0.07	− 0.17	0.06	0.68**	0.23	–		
15. Number of adverse childhood experiences	− 0.05	0.58**	0.38*	0.30^+^	0.23	0.02	− 0.01	0.13	0.07	− 0.06	− 0.26	− 0.18	− 0.11	0.07	–	
16. Warm parenting^a^	0.16	0.15	0.18	0.12	0.13	0.08	− 0.14	− 0.09	− 0.34*	0.29^+^	− 0.26	− 0.50**	− 0.11	− 0.31^+^	0.05	–

Spearman partial correlations, controlling for child age and sex.* PR*  discrimination accuracy,* BR* response bias. *df* = 38. ^a^*df* = 36. ^+^*p* < 0.10, *p < 0.05, **p < 0.01

### Associations between emotion recognition and adopted children’s psychological adjustment, pre-adoptive experiences, and post-adoptive parental warmth

Agreement between parent and teacher ratings of adopted children's emotional and behavioural problems was assessed using raw item scores for the emotional and behavioural scales. There was significant agreement between parents and teachers in their ratings of children’s emotional (average *ICC* = 0.69) and behavioural (average *ICC* = 0.58) problems (both *p*s < 0.001). We investigated associations between adopted children’s facial emotion recognition scores and their parent and teacher-rated emotional and behavioural problems. Given that differences in emotion recognition were detected as a function of sex, where females (*M* = 0.61, *SD* = 0.25) outperformed males (*M* = 0.44, *SD* = 0.25) in discrimination accuracy of angry faces (*p* = 0.03), and child age was positively associated with discrimination accuracy of scared, angry, and neutral faces, *r*_s_s(38) = 0.31 to 0.38, *p*s < 0.05, child sex and age was controlled in all analyses. No significant associations were detected between discrimination accuracy and response bias and children’s receptive vocabulary (all *p*s > 0.05), and so was not included in subsequent analyses.

#### Children’s emotional and behavioural problems at home and school

With age and sex controlled, lower accuracy in discriminating angry and scared faces were associated with higher parent, *r*_*s*_ (38) =  − 0.35, *p* = 0.03, and teacher ratings of emotional problems *r*_*s*_ (38) = − 0.38, *p* = 0.02, respectively. Less accurate discrimination of scared and neutral faces was associated with higher parent-rated behavioural problems, *r*_*s*_ (38) = − 0.45, *p* = 0.004 and *r*_*s*_ (38) = − 0.41,* p* = 0.01, respectively. We found no significant associations between adopted children’s response bias and parent and teacher ratings of emotional and behavioural problems (all *p*s > 0.05, see Table 3).

#### Children’s pre-adoptive history of adversity

We also investigated associations between adopted children’s early experiences of adversity and their facial emotion recognition scores. Children who had more adverse childhood experiences had higher discrimination accuracy for negative emotional faces, including sad, *r*_*s*_ (38) = 0.58, *p* < 0.001, and scared, *r*_*s*_ (38) = 0.38,* p* = 0.02, expressions (see Table 3). No significant associations were detected between pre-adoptive adversity and response bias scores (all *p*s > 0.10).

#### Warm parenting

Table 3 shows associations between parental warmth and children’s facial emotion recognition and parent- and teacher-rated emotional and behavioural problems. Higher ratings of parental warmth were associated with a lower tendency for children to show a bias towards labelling faces as angry, *r*_s_ (36) = − 0.34, *p* = 0.04. Additionally, children were rated by parents to have fewer behavioural problems where parental warmth was high *r*_s_ (36) = − 0.50, *p* = 0.002. Although only trending toward significance, this association was similar for teacher-rated child behavioural problems *r*_s_ (36) = − 0.31, *p* = 0.06.

## Discussion

We investigated emotion recognition in a sample of children adopted from local authority care in the UK. Our findings corroborate previous work demonstrating that typically developing young children and children referred for emotional and behavioural problems are more accurate in identifying happy facial expressions than other basic negative emotions (sadness, fear, anger) [23, 32, 71]. We found similar patterns in performance in both the adopted and non-adopted samples. However, compared to non-adopted children presenting with similar levels of problem behaviours, adopted children performed significantly worse in their discrimination accuracy of sad and angry faces. Contrary to our expectations, we did not detect any differences between groups in response bias towards angry faces.

Although it is well established that impairments in emotion recognition in childhood are associated with emotional and behavioural problems, e.g., [23, 32, 72], to our knowledge, this study is the first to examine discrimination accuracy and response bias in recognising emotional facial expressions in children adopted from local authority care in relation to their emotional and behavioural problems. Lower accuracy in discriminating scared and neutral faces was associated with more behavioural problems rated by parents, which aligns with the Integrated Emotion Systems model (IES; [16]), which suggests that impairments in sensitivity to distress cues, such as fearful faces, may hinder a child’s ability to learn to inhibit aggressive behaviour. We also found that children who were less accurate at discriminating negative expressions (angry and scared faces) were rated as having more emotional problems by parents and teachers. Quite possibly, a child’s difficulties processing emotional cues may lead to a cycle of unsuccessful social interactions which may result in social isolation and evoke negative emotions in the child, such as anxiety and loneliness [73, 74]. Although causality cannot be established in this study, evidence suggests that emotion-based interventions may improve children’s ability to recognise emotional expressions and reduce their emotional and behavioural problems [62, 63, 75]; however, future studies are needed to determine the effectiveness of such interventions for children adopted from care.

As a sample, adopted children underperformed in discrimination accuracy of some negative emotions, yet contrary to our expectations, a greater number adverse childhood experiences were associated with better accuracy in discriminating sadness and fear. Possibly, children who experience more pre-adoptive negative emotional situations (e.g., experiences of maltreatment, exposure to household dysfunction, parent mental health problems) may have a greater sensitivity to the perception of negative emotions, which may have presented an adaptive benefit when children resided in an environment where the probability of threat was high [30]. Indeed, although maltreated children are less accurate in identifying facial emotions compared to non-maltreated children [35, 76], they show a heightened perceptual sensitivity to negative facial expressions, such as anger [30, 34]. For example, children with a history of physical abuse appear to have emotional processing abnormalities specific to anger, such as rapid orienting to and delayed disengagement from angry faces [77]. Additionally, young boys of parents with a history of depression show enhanced perceptual sensitivity to sadness [78]. As pointed out by Pollak and colleagues [79], although these processing differences may be adaptive within atypical social environments, the crucial aspect is the child’s flexibility and control over these processes, and how this translates to their self-regulation and behaviour in normative social settings. Research is needed to elucidate these processes to inform therapeutic intervention strategies. Additionally, this study did not capture all aspects of early adversity that may affect a child’s ability to recognise emotions, and therefore these findings should be taken as preliminary. Further work within larger samples where severity, duration, and type of early adverse experiences may be disentangled would elucidate mechanisms by which early experience influences adopted children’s emotion recognition.

Adoptive parents’ warmth during the Five Minute Speech Sample was associated with fewer parent- and teacher-rated behavioural problems, corroborating earlier work using self-report measures that warm parenting is associated with a marked reduction in adoptees’ later problem behaviours [9]. Warm parenting was also associated with a lower tendency for children to exhibit a hostile bias (i.e., incorrectly identifying non-angry expressions as angry) during the emotion recognition task. Our findings align with previous work demonstrating positive associations between positive parenting practices, such as warmth and support, and children’s emotion recognition skills [32, 57, 58]. Warm parenting may provide children with a safe emotional climate by which to experience positive and negative emotions. It is also quite possible that warmth covaries with other aspects of parenting that are important for socialising emotional competence. For example, it is well established that caregivers’ use of mental state language fosters emotion understanding in typically developing children, e.g., [80]; recent work has also identified similar links in a sample of post-institutionalised children [81]. Although the use of an independent measure of the emotional climate of the parent–child relationship (the Five Minute Speech Sample) is a strength of the present study, further studies that harness longitudinal and observational methods to study parent–child interactions would extend this work and better elucidate mechanisms by which features of parent–child interaction influence emotion recognition. Furthermore, such studies may consider examining other, less well studied, features of emotion recognition beyond facial expressions, such as the auditory aspects of emotion recognition, or, affective prosody recognition [82]. Nonetheless, our findings indicate that interventions that foster warm parenting behaviours may be a promising avenue by which adopted children’s emotion recognition skills and emotional and behavioural problems can be improved.

## Limitations

There are some limitations to this study. Our findings are based on small samples, and therefore some effect sizes, for example, the association between pre-adoptive adverse childhood experiences and discrimination accuracy of angry faces, though not statistically significant, were not negligible. The small sample sizes also precluded us from analysing associations with individual types of early adversity (i.e., neglect, maltreatment) that may differentially affect emotion recognition [35]. It is also important to note the limitations of using cumulative adverse childhood experiences as a marker of pre-adoptive adversity. Rather than approaches that treat individual childhood adversities as equal, future studies must consider the duration, severity, and type of experiences and their impact on children’s emotional competence and mental health [83, 84]. Additionally, such work should consider a child’s experiences following their removal from their birth family, such as the time in care and instability in living arrangements. Our results should be taken as preliminary and studies that better capture the complex pre-adoptive history of children adopted from public care in relation to emotional development would be a fruitful direction for future research.

## Conclusions

Notwithstanding its limitations, this study suggests the importance of using tailored assessment to inform targeted intervention strategies that can be harnessed to improve adopted children’s outcomes [1, 2, 13]. Although there are promising interventions that support emotion recognition and improve behavioural outcomes in childhood, e.g., [63], further research must examine whether adopted children may similarly benefit from such interventions. This study builds on evidence that warm parenting has a positive influence on adopted children’s mental health [9]. and extends this by indicating that warm parenting is positively associated with children’s ability to perceive emotions. As such, support of warm parenting is a promising avenue that may foster adopted children’s recognition of emotions and improve their ability to successfully navigate the social world.

## Data Availability

Data are available from the corresponding author upon reasonable request.
